# Dementia-friendly communities: The involvement of people living with dementia

**DOI:** 10.1177/14713012211073200

**Published:** 2022-01-28

**Authors:** Elspeth Mathie, Arthur Antony, Anne Killett, Nicole Darlington, Stefanie Buckner, Louise Lafortune, Andrea Mayrhofer, Angela Dickinson, Michael Woodward, Claire Goodman

**Affiliations:** 3769 University of Hertfordshire, Centre for Research in Public Health and Community Care, College Lane, Hatfield, Hertfordshire, UK; 6106 University of East Anglia, Faculty of Medicine and Health Sciences Norwich, Norfolk, UK; 8018 University of Hertfordshire, Centre for Research in Public Health and Community Care, College Lane, Hatfield, UK; 2152 University of Cambridge, Cambridge Public Health, Forvie Site, Robinson Way, Cambridge, UK; 5994 Newcastle University, Population Health Sciences Institute, Newcastle Upon Tyne, UK; University of Hertfordshire, Centre for Research in Public Health and Community Care, Hatfield, Hertfordshire, UK; University of East Anglia, Faculty of Medicine and Health Sciences Norwich, Norfolk, UK; University of Hertfordshire, Centre for Research in Public Health and Community Care, College Lane, UK

**Keywords:** dementia, involvement, dementia friendly communities, people living with dementia, public engagement

## Abstract

**Background:**

Dementia Friendly Communities (DFCs) offer an approach to community engagement to improve the lives of people living with dementia and their family supporters. The involvement of those living with dementia is key to creating successful DFCs. This paper examines how people affected by dementia were involved in developing and designing DFCs in England, and the impact of their involvement.

**Methods:**

This study used a mixed method case study design in six DFCs in England. Data collection involved documentary analysis, a survey, and interviews and focus groups with service providers and people living with dementia and their supporters.

**Findings:**

All six DFCs aspired to involve people living with dementia and their family supporters, but often relied on a small number of people living with dementia. The range of involvement activities in DFCs included Steering Group meetings, wider public consultations, and enabling feedback through data collection methods such as surveys and ‘ad hoc’ conversations. Organisations within the DFCs with experience of public consultation offered structured opportunities for involvement. There was no evidence of people living with dementia initiating or co-leading the organisation, its direction and/or the activities of the DFCs.

**Conclusion:**

The involvement of people living with dementia in DFCs went beyond rhetoric, with some evidence of context sensitive and meaningful participation. Approaches towards involvement should focus on involvement in strategic planning, and on harnessing expertise in delivering different involvement activities to optimise participation of a greater breadth of people living with dementia. Engagement with local organisations who work with, and for, people living with dementia, and dedicating the resources needed for involvement work, are crucial for creating DFCs. The success of DFCs are determined by how the needs of people living with dementia are identified, discussed and reviewed by those within the community who are most affected.

## Background

Dementia is a global public health priority (WHO, 2017). Over 90% of the OECD (Organisation for Economic Co-operation and Development) countries have some form of Dementia Friendly Community (DFC) initiative (OECD, 2018). DFCs are one of a range of initiatives that aim to improve the lives of people living with dementia and their supporters and to reduce stigma (DoH, 2012). DFCs are defined as ‘a place or culture in which people with dementia and their carers are empowered, supported and included in society, understanding their rights and recognise their full potential’ (ADI, 2016, p. 10). DFCs recognise the imperative of including people living with dementia as valued members of their local communities (Hebert & Scales, 2019; Lin, 2017; Shannon, Bail, & Neville, 2019). However, there are marked differences in how DFCs have been defined and implemented (ADI, 2017; WDC, 2020). The English DFC approach has a formal recognition process with pre-determined criteria and standards, whilst other countries have a less formal approach. England is one of the few countries that has incorporated the creation of DFCs into policy (Buckner et al., 2019a). The former Prime Minister’s Challenge on Dementia 2015 in England (DoH, 2012) called for the creation of DFCs, and the target of half the population to be living in a DFC by 2020 has been exceeded (DoH, 2016). The 2020 Prime Minister’s Challenge and policy targets (DoH, 2016) also aimed to support businesses to work towards becoming dementia friendly and deliver Dementia Friend’s training to an additional three million people. An initiative that partnered with national charity, Alzheimer’s Society, to support dementia awareness.

An assumption of organisations advocating DFCs is that people living with dementia, carers, family members and supporters are involved in designing and shaping DFCs (BSI, 2015). National and international frameworks and guidance is available for those seeking recognition towards becoming dementia friendly. All guidance emphasise the importance of including those living with dementia in the organisation and operation of DFCs (ADI, 2016; Alzheimer’s Society, 2014-5; BSI, 2015; DEEP, 2015). One review highlighted the value people living with dementia can bring to a DFC: they have local knowledge, can identify the barriers and opportunities and have expertise through experience (Blood, 2017). DFCs thus engage with people living with dementia as active agents, participating as citizens and contributing to society (Bartlett & O’Connor, 2007; Birt, Poland, Csipke, & Charlesworth, 2017).

A limited number of evaluations of DFCs have been carried out, though most are descriptive and have focused on a single initiative (Blood, 2017; Williamson, 2016). Scoping work of 100 DFCs found evidence of consultation with people living with dementia, but the extent and nature of the involvement were not described (Woodward et al., 2019). This paper draws on case study findings from the National Evaluation of DFCs conducted between 2017 and 2019, the DEMCOM study (Buckner et al., 2019a; Darlington et al., 2020; Woodward et al., 2019). DFCs in England are context sensitive and evolve differently, and there is no expectation that to be effective they should follow a particular path (Dean et al., 2015). This paper explores how people living with dementia were involved in their DFCs, and their observed impact on DFC organisation and work. The first phase of our DEMCOM study reported that only around one-fifth of dementia-friendly communities actively involved people living with dementia in their establishing, running and monitoring of activities and initiatives (Buckner et al., 2019a).

In our study, we made the distinction between being actively involved in the decision-making and the organisation of DFCs and attending or participating in activities organised by a DFC (as participants, users or consumers of a DFC). The term ‘People Affected by Dementia’ (PAD) is used to include people living with dementia (PLWD) and those who support them day to day. The main focus of this paper is people living with dementia and their role in the development and delivery of DFCs.

## Methods

This paper presents findings from six case studies on whether people living with dementia were reported to have been involved in their DFCs, how this was achieved, who was involved, the involvement approaches used by the DFCs and the impact. Examples for involvement and challenges are highlighted to build knowledge for practice development. The six DFCs across England were purposively selected as case studies from 100 DFCs that were the focus of a first scoping phase (Buckner et al., 2019a). The research approach moved from a desk-based on-line scoping of publicly available DFC material to contact with people living within DFCs. A short list of 33 eligible sites based on different sizes and locations, diversity of DFC approaches, underpinning values, demography and distinctive features (organisation, funding) were invited to express an interest in taking part. Thirteen of the 33 expressed an interest in participation that led to the final selection of case studies.

Each participating DFC was treated as an individual case study with the boundaries defined by those within each DFC. Case study method was used to organise data collection and analysis. Data collection involved interviews, focus groups and observation (DFC meetings), documentary analysis (DFC strategies, Steering Group minutes, leaflets, promotional material, etc.) and a survey. The interviews were carried out with representatives from local organisations and individuals working within each DFC (practice-based stakeholders) as well as people affected by dementia. Documents, interview and focus group data were entered into NVIVO (QSR, 2018) and coded to the thematic categories of the DEMCOM DFC evaluation framework (Goodman et al., 2020). This framework built on a tool developed for appraisal of Age Friendly Cities (Buckner et al., 2019b). The DEMCOM DFC thematic categories were the basis for establishing a DFC, its leadership and governance, DFC activities and environments, monitoring and evaluation, and resources. Cross-cutting themes were the involvement of people affected by dementia, equalities and inclusion, and evolution of the DFC. Data were first mapped and analysed within case and findings were compared across thematic categories and cases.

The terms engagement and involvement are often used interchangeably. In this paper, we distinguish between engagement, largely the provision and dissemination of information, and involvement, where people affected by dementia are actively involved in shaping and designing research (INVOLVE, 2012) or services. Our analysis of involvement activity was guided by previous patient and public literature on ‘levels’, ‘ladders’ (Arnstein, 1969) and frameworks of involvement. In 2012, INVOLVE (a national advisory group) described three approaches to involvement, which are consultation, collaboration and lay control (INVOLVE, 2012) and the more recent emphasis on co-production (and sharing of power) (Beresford et al., 2021; Hickey, 2018). Although, Oliver et al. produced their conceptual framework of involvement some time ago, it is useful as it is based on three critical dimensions: whether people are involved as individuals or members of organised groups, whether public involvement is at an invitation or a response to action by the public and lastly, the degree to which the public was involved (consultation, collaboration or lay control [Oliver et al., 2008]). Our analysis explored how DFCs reported involvement and how this was enacted in DFC policy, activities and ongoing involvement with people affected by dementia.

Consistent with the topic explored here, involvement of people living with dementia and their supporters (Patient and Public Involvement [PPI]) in the conduct of our study was achieved in the following ways: 67 people living with dementia and their supporters were involved in 35 separate activities throughout the DEMCOM research study. This included representation on the Advisory Committee, participation in a stakeholder event that reviewed early findings, supporting data collection at the case study sites, and commenting on data analysis. A family carer (JT) was also part of the DEMCOM research team.

### Ethical approval

The National Research Ethics Committee (REC) London Queen Square provided ethical approval for this study (ref: 17/LO/0996). Consent was sought from all participants.

### Findings

The six case studies aimed to include a range of examples of DFCs, different sizes, a geographical spread across England, rural/urban DFCs, diverse DFC approaches, with different organisational structures and ranging in start date from 2012 to 2015. The extent of involvement of people affected by dementia in the case studies ranged from one or two people to larger consultations with over 50 people. However, accurate figures were difficult to achieve because DFCs were not routinely recording this information. The way the six case study DFCs had evolved, and the basis on which they had been initiated, influenced the way people affected by dementia were involved. Table 1 provides an overview of the data collected and the sample size for each element of the study in the six case study sites.

**Table 1.** Overview of the participants in DEMCOM and sources of data.

Data source		Site A	Site B	Site C	Site D	Site E	Site F	Total
Doc evidence (*n*)		23	106	47	31	70	27	304
Survey		73	22	9	78	35	23	240
Interviews	PBS	11	8	8	8	9	9	53
	People affected by dementia	1	0	0	0	6	0	7
Focus groups : Participants (number of groups)	PBS	0	0	8 (1)	8 (1)	7 (1)	0	23 (3)
	People affected by dementia	14 (2)	3 (1)	8 (2)	16 (2)	12 (1)	6 (1)	59 (9)
Other (field notes from meetings/events)		5	9	2	1 (4 day visit)	3	1	21

PBS = Practice-based stakeholders (those representing local organisations and individuals working within DFC).

There was some evidence of people affected by dementia being involved at strategic levels, for example: at decision-making level (DFC Steering Groups) and shaping the DFC strategy, shaping dementia activities (e.g. dementia cafes), changing existing services (e.g. carers support), organisations (e.g. transport, shops and cinemas), environment (e.g. signage and access), and monitoring, evaluation and feedback. We are using the term ‘shaping’ to include the range of reported involvement activities from deciding, suggesting, prioritising, modifying, verifying, co-producing, monitoring, feeding back and evaluating DFCs. The findings are organised into four sub-sections: 1) intention to involve, 2) who was involved, 3) involvement approaches and 4) impact of involvement.

## Intention to involve

There was an intention to involve people living with dementia in the planning and design of DFCs in all case study sites. Evidence of intention to involve was found in the strategy documents and plans:

“*There is an underpinning requirement that the voices of people living with dementia are heard and can influence the definition of a DFC and how it is realised.”* (Case Study B: Documentary Evidence: DFC Internal Review)

*“the strategy is informed by what people have told us about their experiences either as a person living with dementia or a carer”* (Case Study F: Documentary Evidence; Dementia Strategy)

The quotes also reveal differing types of involvement in their language ‘underpinning requirement’ and ‘informing’ strategy. There was no evidence of strategy documents being co-authored with people affected by dementia. Involvement often relied on people affected by dementia providing feedback on existing plans, rather than starting with ideas from people affected by dementia:

*“we are currently developing a joint three year plan which we have recently asked stakeholders, including patients and carers to feedback on”* (Case Study E: Documentary Evidence: DFC document)

Local prevalence of dementia was the starting point for each DFC, but our documentary review and interviews found minimal evidence of systematic assessment of the local need and possible priorities. There is a difference between identifying a general need for a DFC and knowing, or assessing, what the local priorities and circumstances are for people affected by dementia. Findings demonstrated that local grass roots initiatives were invariably driven by a general recognition of need, and the stigma and difficulties people affected by dementia were likely to experience. Less evident was how this was operationalised. In one case study, participants were aware that this was missing:

“*what we need is a little more assessment of what the need is, rather than rushing in providing services first, and then saying, ooh, is there a need for it…….it has been rushed into..*….*real ground swell of enthusiasm*” (Case Study E: Focus Group: Dementia Friends Steering Group member)

and another participant continued:

*“we need to know where they are, what they want, and we’ve got to be not just dementia friendly, but aware as a community, and it’s how we do that that’s the problem”* (Case Study E: Focus Group: DFC Coordinator)

### Identifying who was involved in DFCs

People living with dementia who were involved in DFCs were identified in their community in different ways. Some DFCs that had concentrated on arranging ‘dementia-specific’ or separate dementia friendly activities had a natural pool of possible recruits, although these were people already involved with DFCs (as recipients of dementia services or activities). Where the DFC had prioritised inclusion and accessibility by involving people living with dementia in everyday activities, the DFC could trigger a more proactive approach to recruitment and the different types of involvement. People affected by dementia could be involved in assessing how the local environment (buildings, signage) and activities were accessible to all by auditing services and participating in group discussions about how services were planned. Of the six case studies, two reported difficulty in identifying people to be involved. We found no evidence of refusals to participate once invited, but sustaining engagement required extra resources and, often, accessible places to meet. Identification and recruitment were not systematic and little consideration was given to equality and diversity. Two case studies reported good links with health (NHS) diagnostic services, which created an early opportunity to publicise the DFC work and invite participation from the database of people who had recently received a dementia diagnosis. However, it seemed they were not able to build this process into the post diagnosis support and signposting offered by the memory services. It relied on staff telling individuals about the DFC activities during a routine appointment.

Those involved in their DFCs included people living with dementia themselves (either as individuals or attached to groups), family members of people living with dementia (with past or current experience) and charity representatives. These four groups or individuals who were involved are discussed in turn below.

i) People Living with Dementia

People living with dementia were involved as members of the DFC Steering Group in four of the case study sites, and the Steering Group had oversight of the direction of the DFC. Specific information about these people was very limited, referring to ‘a couple’ or ‘one person living with dementia’. The lack of information was revealing and sparse when compared to information about other members of the Steering Group and who they represented (emergency services, local authority and local businesses). This evidence suggested that many of these interactions between DFC Steering Group and people affected by dementia were opportunistic, based on an individual’s interest to join the group and ability to access meetings and contribute. Two case study sites revealed evidence these DFC Steering Group members were representing, or feeding back to, other people living with dementia in the local area. The following quote highlights a positive account of how people worked together:

*“They’re a group of people who are actually living with dementia, but who are actively involved in shaping agendas and participating. You would have no idea, actually – they are like colleagues – until you were told”* (Case Study F: Interview: Local Authority)

This quote reveals two issues; firstly, attitudes and expectations as to what a person living with dementia could contribute and secondly, it suggests that there may not be a general awareness of the need to offer extra support to all for effective participation.

ii) Working with Groups of People Living with Dementia

We encountered two very different relationships with existing groups of people living with dementia. DFCs working with groups where people affected by dementia already attend made it easier for them to be involved in DFC development. One DFC centred around a physical ‘dementia friendly’ building where activities for people living with dementia were held. DFC meetings took place in the building and a small number of people living with dementia regularly attended. In a second site a pre-existing group of people affected by dementia (which had 18 PLWD and their family supporters) challenged the expectation that they should attend DFC meetings. Instead, they proposed a different way of working and invited a representative from the DFC group to come to their group:

“*so we’ve kind of said to the [DFC], “would you come to [our group], rather than* *have lip* *service person join the Steering group…….*“*if you do want some people to come to your […] meetings, you’re going to have to change….you can’t have a normal business meeting*” (Case Study B: Interview: Dementia Group Coordinator)

This model of a parallel organisation was useful in enabling people’s views to be included within an existing group in a safe and familiar environment. It is an alternative (‘reaching out’) model rather than expecting people to fit into the format of an existing formal Steering Group.

iii) Family Supporters, Carers

Family supporters’ involvement could also ensure that the lived experience of dementia was considered, but case study sites recognised the need to differentiate between what was important to people living with dementia versus what was important to their carers. For example:

“…*so she puts across his point of view and also her point of view as a carer and we could do with more people like that but again it’s attracting those people”* (Case Study C: Interview: Dementia Friendly Community Member)

*“there are a few key players that were carers many years ago. I find the danger with that is that they’re very opinionated on what things were like five, 10 years ago and they still consider themselves the experts….but they are listened to because there’s not that many carers who engage with these sorts of things”* (Case Study E: Interview: Specialist Nurse)

Deterioration in health can affect the ability of people to be involved. Inevitably for some of those who had been involved, ‘*dementia has progressed*’ (Case Study C: Interview: Staff Public Health) and this also affected their carers who could no longer leave their family member:

*“he [person living with dementia] used to come along [events committee] but he’s not as good as he used to be and so he can’t cope with that”* (Case Study C: Interview: Dementia Friendly Community Member)

*“there is always going to be a bit of a challenge with engaging people because of the nature of the condition”* (Case Study C: Interview: Retired Nurse)

When working with groups of people living with dementia, there will inevitably be a turnover of people who can be involved for as long as they are willing or able, but also a realistic requirement to encourage new people to become involved. This reiterated the need for DFCs to maintain connections with a wide range of people. Involvement often relied on people being self-selecting and motivated to join an existing committee or panel. Participation and engagement were sought but often underdeveloped:

*“we don’t actually have the people sitting round the table very often at all now who are living with dementia, and that is probably the biggest challenge”* (Case Study C: Interview: Public Health)

iv) Charities or Third Parties

Lastly, there were also examples where peoples’ views were represented by charities or third parties:

*“Q: do you work directly with any people who are affected by dementia in the sense that they shape the activities and projects that you are providing?”*

*“A: Not directly, no. I would say no, we work with organisations like the Alzheimer’s Society on the whole and carers organisations, but not directly with people living with dementia”*(Case Study E: Interview: Fire Prevention Officer)

In many of the DFCs, people affected by dementia were also users of the DFC services, and this was particularly true if the basis of the DFCs focussed on dementia specific activities. In these situations, the relationship was more akin to engagement than involvement, as consumers providing feedback about existing activities or services rather than planning from the outset. Over the course of the study, there was some evidence of how participation in a DFC changed individuals’ expectations and attitudes. Involvement in DFCs meant individuals started to recognise their rights, and that they should not have to accept, for example, inaccessible public transport:

*“I don’t use buses very often, but when I use them, I say to the bus driver ‘Can you wait a minute?’ I just shout at them, I go ‘Right, we’re okay’ and I make them aware it’s going to take us a little bit longer to get sat down and that”* (Case Study D: Focus Group: Person affected by dementia)

Implicit in the awareness raising work and collaboration with local organisations and their systems of working was recognition of the need to be inclusive. None of the DFCs had an explicit rights-based agenda in how they described their organisation and worked. We suggest that without a rights-based agenda (or at least a raised dementia awareness and the development of a vocabulary to name and voice an experience), it was harder for individuals living with dementia to organise around a collective view, or narrative, of what they had the right to expect post diagnosis (Hagan, 2020).

### Involvement approaches

There are a wide range of practices that aim for marginalised and disenfranchised groups’ voices to be heard that have become mainstream in some statutory organisations (SCIE, 2015). Some DFC participants working, for example, in local authorities were familiar with the aspirations of these practices, such as community engagement; ‘co-production is the buzzword within adult social care at the moment, so with all of our commissioning, we are supposed to be

*co-producing with service users appropriate service users and their carers at all points of the commissioning cycle. So there will be plenty of scope for engaging with people affected by dementia and their carers’* (Case Study F: Interview: Local Authority)

However, neither documentary analysis nor interview data indicated any examples of people living with dementia leading their DFC. There was no evidence of joint decision-making or co-production but many examples of consultation and engagement. There were opportunities to tap into existing groups with growing memberships of people living with dementia, and this offered the opportunity to develop a collective voice and a more sustainable approach.

A wide range of approaches were found in the case study sites where people affected by dementia had been involved in shaping DFCs. This included membership on Steering Committees (discussed in ‘Identifying who was involved in DFCs’ section above), public consultations, acting on individuals’ feedback about their experiences as well as more formal ‘research methods’ such as surveys and focus groups to gather views and opinions (see Table 2). Table 2 presents the range of approaches, the benefits and challenges of using these methods, and an estimate of the number of people who were involved. There was no systematic or accurate recording of numbers of people who attended consultation events, who they represented, and their access to different levels of support. It was noted that there was also a mixture of formal involvement through planned activities but also opportunities for informal, unplanned involvement through conversation at events.

**Table 2.** Examples of involvement activities found in DFCs.

Level of involvement	Involvement approach	Considerations raised	Numbers involved^a^
Strategic	Member of steering committee (working group of DFC)	People affected by dementia tend to be mobile, confident in meetings, same people often involved in several committees	Small number (usually 1 or 2)
Strategic or consultation	Parallel organisation Steering committee members attend a pre-existing dementia involvement group that meets regularly and has funded support	Group already have rapport amongst themselves, and support. Safe environment, people already know each other and able to engage with a range of issues	Larger numbers (15–30)
Consultation	Organised public consultation event Informal word of mouth	Easy to organise, try to aim for diversity of group, reliant on key people promoting participation	Larger numbers (30+)
Consultation	Listening at existing dementia groups (e.g. Dementia Café)	People living with dementia or supporters speaking on behalf of who? Different needs Only involves those already engaged in dementia activities	Larger numbers (30+)
Consultation	Seeking feedback through surveys and focus groups	May need support for people affected by dementia to take part, need to offer a range of methods (face to face, paper and on-line). Questions sent ahead of time, on the spot consultations might not be so good for people living with dementia Early consultation has potential to shape DFC more	Larger numbers (can reach a more diverse population) (Darlington et al., 2020)
Monitoring and evaluation	Audit, mystery shopping	Organising audit needs support. Providers can get immediate feedback	Small numbers, but potential to include a larger group of people. Achieves measurable impact on service

Key

^a^approximations.

People affected by dementia = People living with dementia and family supporters

Mystery shopping = People living with dementia make unannounced/anonymous visits (to shops, buildings) and feedback on interactions with staff, accessibility, signage, etc.

### Public consultations

Consultation events occurred in all the sites and targeted people living with dementia; however, the term was used very loosely. Consultations were held to share DFC strategies and invite comment on proposed and current services. The consultations were sometimes held in public spaces face to face, whilst some DFCs consulted with existing groups. For example, one site held an initial meeting for 50 people living with dementia, followed by another consultation in a community setting (a football stadium) involving people from Black Asian and Minority Ethnic (BAME) communities. This consultation included statutory agencies, Public Health, the NHS Foundation Trust, Clinical Commissioning Groups and local community representatives. It was one of the few consultations that aimed to include people from specific communities. Another case study had a BAME specialist dementia worker post. In another example, consultation was used to recruit people for future involvement:

*“through the consultation around the dementia strategy, one of the questions we asked was if people wanted to get involved in future work around dementia or carers, to provide us with their e-mail address. We had I think 50-odd city residents that were interested in becoming involved in work. Now, a lot of those people were carers, 23 of them were carers of people affected by dementia, but I think that’s a good thing. If it’s not people affected by dementia themselves then it’s carers of people affected by dementia, and that’s fine because they can do the talking for us with the person themselves”* (Case Study F: Interview: Local Authority)

How the consultations were acted upon was not always clear, as in this description of listening:

*“the project began by listening to what people with dementia and carers thought would make [site x] more dementia friendly”* (Case Study D: Documentary Evidence: Progress to date: consultation)

However, there were also specific examples of actions (e.g. changes to physical environment, transport) that could be tracked back to these consultation events, although their main function was to raise awareness across the DFC and initiate conversations that could lead to closer working.

The importance of the local authority, or of people knowledgeable about public engagement/involvement, was evident in the sites who ran public consultations or community engagement events (not necessarily dementia specific ones). They were able to draw on resources of expertise and people, and to exploit their knowledge of local networks.

### Listening events and surveys

‘Listening’ events (described as being similar to consultations but more open ended, not focussing on a specific item), or going to existing dementia services to talk to people, were other means of informing DFC start up and planning. These could be supplemented by surveys aiming to capture local people’s priorities, environmental adaptation needs, and feedback on existing activities run by the DFCs. One respondent recognised the time and resource required to consult people properly, and that people living with dementia need extra support to participate meaningfully:

*“…can’t just show up there; you do have to plan that in, because you have to be able to send them out with the questions you want to ask them beforehand, so they’ve got time to sit and think about it rather than expecting an immediate response”* (Case Study F: Interview: NHS nurse)

### Monitoring and evaluation

Three of the case study sites involved people living with dementia and their supporters in monitoring and evaluation of their local community and services through auditing or mystery shopping:

*“So if an organisation like, say, the leisure centre joined [the DFC], then this couple will go and do an audit, and go in and do an environmental audit to make sure that the signposting is clear, that they can navigate their way around the building, and make suggestions as to what signposting needs changing. So we try to keep them as involved as we can”* (Case Study C: Interview: Dementia Friendly Community Member)

The importance of ongoing involvement was mentioned by one participant:

*“I think with dementia being as it is on the journey, just revisiting that customer journey along the way as well, because that customer journey will change as the dementia progresses. So it’s important to keep going back and keep asking. It’s absolutely no good asking once or twice. You’ve got to keep going back and asking”* (Case Study D: Focus Group: Dementia Friendly Champion)

Overall, involvement approaches and subsequent impact ranged from working with self-selected people able to attend meetings, to trying to engage with the whole community at all stages of the dementia trajectory using a variety of different approaches.

### Impact of involvement of people affected by dementia on their DFC

To support future evaluations, the study wanted to develop a way to assess the extent of involvement of people affected by dementia to support learning and development. However, it was difficult to capture how people living with and affected by dementia had influenced the priorities and basis of the DFC. It was unclear if acting on their input was seen as obligatory by the DFC leaders and who were considered ‘the experts’.

*Q: So before any initiative comes about or any kind of activity starts, do people speak to people living with dementia first or are they involved in any way*?

*“A: Well, not as much as I would like, no. Hands up, I’d have to say no. What we do is take advice from the experts [DFC Steering Committee] and then implement those changes based on what they tell us”* (Case Study F: Interview: Retail Sector)

There were a few concrete examples of how those involved knew their input had made a difference: where people living in their DFCs were involved in local decisions on individual activities, services or organisations. For example, there was evidence of people living with dementia and their supporters affecting campaigns and changing service responses when there was personalised feedback and action on specific issues;

‘*and I do know that following that [survey] the group approached supermarkets about creating maybe dementia-friendly slots for people to go and do their shopping, or just exploring whether they could change the way that tills were operated*’ (Case Study E: Interview: Charity)

The importance of continual involvement to ensure impact was emphasised in one site with an example of a service that discontinued after feedback was received:

“*everything we do at all is driven by the people who come to the group, so if they say actually this isn’t what we want to do, we’d rather do this, then that’s what the funding goes toward*s*....we do follow through if people say yes, I’d like to do that*” (Case Study A: Interview: Charity)

This quote views people living with dementia as consumers of services rather than partners. In one case study site the DFC coordinator was proactive in seeking views from people in the community and attempting to achieve direct change as a result. For example, creating dementia friendly car parking spaces following a conversation with a couple affected by dementia and providing feedback to taxi firms and bus drivers when customers reported that their service was inclusive and responsive.

Where consultations were an iterative process, continually feeding into the DFC, this supported dialogue and informal evaluation of what worked. In one case study site, at the end of a dementia friendly cinema screening immediate feedback was sought by a simple thumbs up, thumbs down and asking for views at the time.

“*when we’ve done the consultation which we’ve done several times, we want people to know what they’ve fed back to us, we’ve taken notice and we’re doing something about it”* (Case Study D: Interview: Public Health)

However, a minority of participants living with dementia felt that their involvement was tokenistic with a narrow sharing of experience:

*“That’s all you’re doing, you’re just there to tick a box and that’s it. Nobody takes people on board to see what they’re going to do and see whether they can have an impact on what is available or what isn’t available. They just want to tick boxes very often, I think. That’s all the councils do, that’s all they’re there to do”* (Case Study F: Focus Group: PLWD)

One person living with dementia reported ‘*it would seem everybody and their brother want a person with dementia on their steering groups*’ (Case Study A: Interview: PLWD). This individual was involved in a number of organisations, sitting on the Dementia Action Alliance, Hospital Dementia Steering Group, the Service User Research Group and was also a Dementia Champion. Consequently, he had learnt to ask before contributing to any further groups (including this study) for evidence of how his involvement could make a difference.

## Discussion

The study findings demonstrated a continuum of engagement and involvement from perceived tokenism to active decision-making on specific aspects of DFC work. Position on this continuum was influenced by prior experience of public consultation, access to support (for people affected by dementia) and both planned and unplanned opportunities (Oliver et al., 2008) to influence the DFC. There was a universal commitment to this involvement process, but also a lack of strategic approaches compounded by lack of resources and expertise in how to support people to participate. Examples of DFCs connecting with existing groups for people living with dementia (creating opportunities for regular feedback and participation) and with active support from statutory providers (e.g. Local Authority and NHS providers) led to shared learning, responsiveness and tailoring of activities. In one instance, the process of engagement by people living with dementia led to an awareness of their right to be recognised and included as valued members of their local community. Overall, the findings suggest the nature of engagement with people affected by dementia was as valued consumers of DFC services rather than partners and opinion leaders. Age Friendly Cities provide useful examples and lessons on how to involve older people through co-creation, co-production and partnership approaches (Rémillard-Boilard, Buffel, & Phillipson, 2017).

The intrinsic right of people living with dementia to be involved in activities and services designed for them draws on an equalities-based approach, social model of disability and disability rights (Shakespeare, Zeilig, & Mittler, 2019). This views people living with dementia ‘as equal citizens with their value recognised and respected’ (MHF, 2015, p. 21). It could be argued that without enacting such a rights-based approach, DFCs have a built in limiting factor by which they are at risk of perpetuating the very stigma and exclusion from society they were conceived to address (WHO, 2015). Even the term ‘Dementia Friendly’ could be seen as paternalistic, rather than referring to communities that are ‘inclusive and accessible for all’ (Rahman & Swaffer, 2018). Whilst in health and social care and research practices the term ‘co-production’ (Hickey et al., 2018; NIHR, 2015), with joint ownership of key decisions, has become increasingly popular, there was little evidence of co-production within DFCs. This raises the question of how DFCs should continue to develop if people living with dementia are not central to their organisation, and creates a tension between what ought to happen and what is achievable (Dean et al., 2015; Hagan & Campbell, 2021; Hare, 2016). Apart from DFCs, there is an increasing body of evidence about what is important to people living with dementia (Reilly et al., 2020). This provides a foundation for involvement work, especially in a context where most DFCs have limited resources to develop plans and continuously monitor outcomes for evaluation of their work.

The participants in this study all felt that involvement was important. The more DFCs involved people living with dementia, the more they became convinced of its value. None of our sites had a mechanism to link with people through memory clinics or their GPs. People report that there is little social support or signposting following a diagnosis of dementia (Hagan, 2020). Sustaining and embedding involvement required both resources and strategies to thread these activities through planning, delivery and evaluation.

The reasons for people living with dementia not being involved was not discussed explicitly within the DFCs, apart from acknowledging it was difficult to identify people living with dementia. Hare (2016) states “Local grass roots community activity is the bedrock of DFCs” and “this activity must be supported by strong strategic planning, commissioning and leadership” (p. 140). At the time of the DEMCOM study, involving people affected by dementia was valued but discretionary. Normalisation process theory articulates core constructs needed for an intervention, such as a DFC, to achieve a shift in thinking and collective actions. In the context of involvement practices and DFCs, these core constructs are coherence (sense making, people recognising the value of improving inclusion and access for people affected by dementia), cognitive participation (building relationships), collective action (those doing the operational work) and reflexive monitoring (appraisal). They point to actions to embed involvement into ‘normal practice’ (Murray et al., 2010).

There are many lessons to be learnt from involving member of the public in health and social care research and services. The approaches described in this paper are not a hierarchy of involvement (Arnstein, 1969; Oliver et al., 2008), rather they capture what is possible in different contexts. Previous involvement models have been criticised for being hierarchical, one dimensional and having a lack of recognition of power imbalances (Carpentier, 2016; Gibson, Britten, & Lynch, 2012) and political context (Beresford, 2002). Current debates around co-production recognise structural issues of involvement and the sharing of power (King & Gillard, 2019; Williams et al., 2020). Offering a range of different ways to become involved will hopefully enable a wider group of people to do so. Where involvement was sufficiently supported and enabled, employing a variety of approaches and means, DFCs were able to demonstrate a more dynamic partnership and iterative approach to involvement. The recently launched Public Involvement Standards in the UK (NIHR, 2019) offer transferable learning opportunities for DFCs (standards include inclusive opportunities, working together, support and learning (Staley, 2015), communications, impact and governance) to monitor their ‘involvement’ progress. Involvement then becomes more than a ‘one-off’ activity, using different community engagement approaches and coproduction (Bortoli, 2021) to achieve impact with involvement embedded throughout (Wilson et al., 2015).

This study demonstrated the value of sharing learning across DFCs of where involvement works well, and challenges are encountered (Dean et al., 2015). For example, DFCs whose organisations had experience of public consultations, and those who worked with parallel dementia groups, provided a template that could have wider uptake. There is much to learn from dementia led initiates such as Dementia Engagement and Empowerment Project (DEEP) and Innovations in Dementia (DEEP, 2015). Also, the growing literature that involves people living with dementia in research provides many successful examples of methodologies to include lived experience (Bethell et al., 2018; Pickett & Murray, 2018). Many of the reasons people are excluded from being involved are relevant here: equality issues, where people live, communication issues, nature of impairments and unwanted voices (Beresford, 2013). People who cannot travel to meetings, live in residential care settings or are later in their dementia journey were invariably excluded from contributing to shaping their own DFCs. It is also important to remember people living with dementia are not a homogeneous group (Kitwood, 1997).

DFCs included a range of voices: the person living with dementia (as an individual or part of an established group), the family supporter and charity representatives. These voices may bring different views (Bartlett et al., 2018). Further work is needed to consider how to interpret the sometimes competing accounts, and achieve involvement that can hold this creative tension for the long-term benefit of people living with dementia.

Our findings suggest a number of recommendations: a more strategic approach to enable a diverse group of people affected by dementia to be involved in their own DFC, DFCs to offer a wider variety of approaches for people to be involved, a link with local dementia diagnosis services, and more sharing of involvement successes and challenges between DFCs. DFCs also need resources and expertise to ensure people are adequately supported to be involved.

Lastly, some significant tensions were identified in the study such as the challenge of finding a balance between working with self-selecting individuals versus the wider population of people living with dementia, the involvement of greater numbers of people versus working in greater depth with a few and the tensions between standardised methods and practices for the benefit of comparison between DFCs compared to the benefits of experimentation and local innovation.

The limitations of our study are that the findings are drawn from a limited number of DFCs and from self-reported involvement. However, these findings corroborate those from the first phase of the DEMCOM study (Buckner et al., 2019a) to provide an in-depth account of what the scoping work had identified. We aimed to include many voices in this research by including people affected by dementia as participants. We recognise, however, that similar to those involved in DFCs, it was difficult to include the diverse range of people who would have contributed additional view and insights.

## Conclusion

As a social movement, DFCs in England have achieved a visible national presence. DFCs have involved people affected by dementia, but to achieve more, they need to work with existing groups or community organisations that involve people living with dementia. They also need to support structured approaches and dedicated resources to engage with, and feedback to, people affected by dementia. This includes investing resources in documenting and recording involvement in ways that support comparison, and track how this is achieved over time. There is a lack of guidance to help DFCs effectively involve people living with dementia. The creation of communities that are dementia ‘enabling’ by those who live in them can lead to a more inclusive community for all.
